# Cross-sectional study of mental health related knowledge and attitudes among care assistant workers in Guangzhou, China

**DOI:** 10.1186/s13033-021-00441-7

**Published:** 2021-02-23

**Authors:** Jie Li, Xiao-Ling Duan, Hua-Qing Zhong, Wen Chen, Sara Evans‑Lacko, Graham Thornicroft

**Affiliations:** 1 The Affiliated Brain Hospital of Guangzhou Medical University (Guangzhou Huiai Hospital), NO. 36 Mingxin Road, Liwan District, Guangzhou, 510370 China; 2 Faculty of Medical Statistics and Epidemiology, School of Public Health, Sun Yat-Sen Center for Migrant Health Policy, Sun Yat-Sen University, Guangzhou, China; 3 Care Policy Evaluation Centre, London School of Economics and Political Science, London, UK; 4 Centre for Global Mental Health, Institute of Psychiatry, Psychology and Neuroscience, King’s College London, London, SE5 8AF UK

**Keywords:** Care assistant workers, Knowledge and attitudes, Stigma and discrimination, Severe mental disorders, Low- and middle-income countries, Human rights

## Abstract

**Background:**

Care assistant workers (CAWs) are a part of a new pattern of mental health care providers in China and play a significant role in bridging the human resource shortage. CAWs in China mainly include community cadres, community mental health staff, and community policemen. The mental health related knowledge and attitudes of CAWs could influence their mental health care delivery. This study aimed to assess mental health related knowledge and attitudes of CAWs in Guangzhou, China.

**Methods:**

In November 2017, a study was conducted among 381 CAWs from four districts of Guangzhou, China. Participants were assessed using the Perceived Devaluation and Discrimination Scale (PDD), the Mental Health Knowledge Schedule (MAKS), and the Mental illness: Clinicians’ Attitudes (MICA) Scale. Data were analyzed by descriptive statistics, ANOVA, Bonferroni corrections and multivariable linear regression.

**Results:**

The mean scores (standard deviation) of PDD, MAKS and MICA were 36.45 (6.54), 22.72 (2.56), and 51.67 (7.88), respectively. Univariate analyses showed that the older CAWs, community policemen and those who were less willing to deliver care to people with mental illness had significant higher MICA scores when compared with other staff (*P* < 0.001). Multivariable linear regression showed that after controlling for key variables, care willingness and PDD total score were positively associated with the MICA total score (all *P* < 0.05), while attitudes on additional items were significant negatively with the MICA total score (all *P* < 0.01).

**Conclusion:**

These findings suggest negative attitudes towards people with mental disorders among CAWs are common, especially among older staff. Community policemen suggest that they applied stereotypes of “violent mentally ill” people to all people they deal with who have mental disorders. The results also indicate human rights are being paid some attention to now, but need to be further continually improved in the future. Strategies for improving such negative attitudes and reducing the perceived stigma and discrimination should be carried out towards particular staff groups in an anti-stigma programme in Guangzhou, China.

## Background

Severe mental disorders (SMD), such as schizophrenia and bipolar disorder, are a main cause of high disability and premature mortality in the world [1]. It is known that mental disorders account for 32.4% of years lived with disability and 13.0% of disability-adjusted life-years and also confers a higher mortality risk [2, 3]. Moreover, there is also increasing evidence suggesting that mental disorders account for more than approximately 13% of the global burden of disease [1, 4, 5]. It is predicted that mental disorders will represent one third of the economic burden of all non-communicable diseases by 2030 [6]. Given that SMD have an influence on well-being of individuals, happiness of families, and harmony of society. Stigma related to mental illness sets barriers to patients’ psycho-social recovery and returning to education, work settings and participation in the community. Effective measures should be taken to deliver better services to people living with SMD [7, 8]. With the development of economy and society in high- income countries, it now more common to see a trend to form a balanced care model between specialized hospitals and community care for improving mental health services in high- income countries [9].

When compared to high-income countries, however, there are more challenges to improve mental health services in low- and middle-income countries (LMICs), such as the huge treatment gap (TG) and shortage of available human resources [10]. The treatment gap refers to the fact that more than 95% of people with major depressive disorder in LMICs receive no effective treatment [11]. The WHO also have reported that 97% of high-income countries deliver community-based care, but the proportion in low-income countries is only about 52% [12]. Due to the fact that mental health care mainly relies on professional staff, rather than advanced technology or medical equipment, the WHO has proposed strategies called task shifting (TS) for increasing human resources in mental health care, which means to shift part of services or roles from mental health staff to non-specialist health workers in the community [1, 13].

China, one of the middle-income countries in the world, has large number of people diagnosed with mental disorders. In 2012, it is reported that 173 million Chinese people were estimated to have been diagnosed as psychiatric disorders, of whom 158 million receive no treatment [14]. However, policies of mental health in China have been developed according to different characteristics in different historical periods. There are three major changes in the period of delivering mental health care in China: The policy of “public prevention and public treatment” adopted in the 1950s for people with SMD, the prevention and treatment management mode dominated by specialized psychiatric hospitals from the 1960s to the 1990s, and a rehabilitation management mode featuring community services combined with hospitals since the 1990s. The Chinese government has taken effective measures to face challenges and address some mental health needs in recent years, including efforts in 2005 to cover psychotropic drug costs in basic health insurance, an initiative in 2010 building of more psychiatric hospitals and psycho-psychiatric units in general hospitals, and the “686 Programme” since 2004 which aims to integrate resources of hospital and community services and to train mental health staff in case management and to use individual care plans [14, 15].

China is experiencing a shift from a model of care focusing on single psychiatric institution to a new combination of multiple specialized hospitals, general hospitals and communities services which results in the emergence of a new types of mental health care providers in communities, named care assistant workers (CAWs). CAWs arise from the specific socio-political culture in China, especially for community policemen who are responsible the local security situation. CAWs mainly consist of community cadres, community mental health staff and community policemen. Among them, community mental health staff are primarily responsible for the diagnosis and treatment of patients with mental disorders in community settings. Community cadres are mainly in charge of providing comprehensive services for people living in the community and help with follow-up care, supervise medication compliance, and the crisis management of patients and caregivers.

Local policemen in the community usually have close contact with SMD patients with unstable psychiatric symptoms or violent behaviour, and they can assist people with mental disorder to go to hospital if needed. For community policemen, there is trend that they are in transition from seeing psychiatric patient as “bad” to “mad” in their work. Community policemen used to be in charge of most criminals and a small proportion were people with mental disorders. Gradually, the scope of their work has expanded, from managing people who have severe violent behaviour to those who have manifest less disturbed behaviour. In this context, community cadres and community policemen are also regarded as lay mental health workers (LHWs) [16]. It refers to those workers who lack a formal medical professional certificate or degree, but who are part of the wider workforce in the field of mental health.

Knowledge and attitudes towards SMD among CAWs can greatly influence their behavior and the quality of services towards patients with SMD. Negative attitudes and discriminatory behaviors could cause adverse consequences, such as unwillingness to deliver care, spending less time with such patients, or disregarding human rights [17]. However, it is still unclear what the current levels of knowledge and attitudes are among CAWs, especially for community policemen. Therefore, assessing these baseline levels of CAWs is crucial to track and improve mental health care, including in Guangzhou which is one of the largest metropolitan cities in China. Guangzhou has its own mental health service model named “PTSA” (Policy, Training, Service, and Assessment) and assessing such baseline levels will be an important part of assessing future service improvement [18].

This is the first study aimed at assessing the current level of knowledge and attitudes among CAWs in Guangzhou, China. We hypothesized that different types of CAWs with different age groups, care willingness would have different level of knowledge and attitudes related to mental health.

## Risk assessment of patients with SMD in China

In 2004, the "686 Programme" was initiated in China to strengthen the management and treatment of people with SMD, which aimed to construct a national community-based system [19]. In this programme, the risk assessment questionnaire was used to assess risk assessment level of people with SMD.

There are six levels in the risk assessment questionnaire, all ranging from 0 to 5. Levels were classified as follows: People in level 0 were those who showed no risks; People in level 1 were those who showed verbal threats and shouting, but no actual damage or assaults; People in level 2 were those who had shown behavior of damage to property, which happened only at home and could be persuaded to stop; People in level 3 were those who had shown repeated damage or attacks on property or people, which happened anywhere and could not be persuaded to stop; People in level 4 were persistent damage or attacks on property or people (including self-injury or suicide); People in level 5 were those who had any behavior such as serious violence against others, whether at home or in public.

Among 50,000 people with SMD registered in the registry system of Guangzhou SMD Management Database, nearly 1000 (2%) of people were assessed in level 3 to level 5 who were those with potential violence to themselves, their families or the wider society. Therefore, CAWs who delivered services towards patients with a level of 3 or higher had priority as participants in this study.

## Methods

### Study design and participants

This cross-sectional study which lasted for nearly a month was led by Guangzhou Huiai Hospital (current name is The Affiliated Brain Hospital of Guangzhou Medical University) and conducted in the community on November, 2017. Ethics application was approved by the Research Ethics Committee of Guangzhou Huiai Hospital (Number 0252017). Based on our previous study [20], 4 of 11 districts were randomly chosen based on their geographical locations in Guangzhou City. Finally, Li Wan and Tian He were included as urban districts; Hua Du and Nan Sha were chosen as suburban districts.

Participants in our study were all CAWs who worked in the four selected districts and delivered mental health services to patients with SMD assessed as at level 3 or higher by the risk assessment questionnaire. We also required that CAWs who participated in this study should be those who were treating service users should satisfy a series of criteria, namely taking antipsychotic medication, being in a stable condition and living in the community for at least half a year. Participants were asked to complete three standardized scales to assess their baseline level of knowledge and attitudes. Thus, this research mainly aims at learning the baseline level of knowledge and attitudes among CAWs. Apart of this baseline study (part 1), we have further developed an anti-stigma training for CAWs in Guangzhou China (part 2), please see a prior publicaition for full details [21]. This project is part of the wider “Anti-stigma campaign in Guangzhou,China” (2017–2022).

After the procedure had been thoroughly explained, participants were those who gave their written, informed consent. A total of 381 participants were included in data analysis.

## Instruments

### Perceived devaluation and discrimination scale (PDD)

Perceived Devaluation and Discrimination Scale (PDD) was developed by Link et al., to assess the beliefs of participants regarding to which extent other people will devalue or discriminate against somebody with mental disorders. The scale consisted of 12 items rated by 5 points from strongly agree to strongly disagree [22]. Total score of PDD ranged from 12 to 60 and item 5, 6, 7, 9, 11 and 12 are reversely scored. The higher score suggested the stronger perception of devaluation and discrimination. In this study, we used the Chinese version of PDD which has been tested with good validity and reliability (Cronbach's Alpha = 0.70) [23, 24].

### Mental health knowledge schedule (MAKS)

The Mental Health Knowledge Schedule (MAKS) was developed by Thornicroft and his colleagues from King's College London, United Kingdom. It is used to measure participants' knowledge and understanding regarding stigma related to mental illness. The scale consists of 12 items, which were rated on the 5-point Likert scale ranging from strongly agree to strongly disagree [25]. The higher score suggested more stigma-related mental health knowledge. MAKS is made up of two parts: Part A (items 1–6) aimed at measuring knowledge and understanding of stigma and discrimination; Part B (items 7–12) aimed at measuring the ability to identify depression, stress, schizophrenia, bipolar disorder, drug addiction and grief. In this study, MAKS was used in conjunction with PDD and MICA. Overall test–retest reliability of MAKS was 0.71 and the overall internal consistency among was 0.65 (Cronbach's Alpha) [25]. The Chinese version of MAKS was introduced by Li from Guangzhou Huiai Hospital [26].

### Mental illness: clinicians’ attitudes (MICA) scale

Mental illness: Clinicians’ Attitudes (MICA) Scale was used to measure the level of stigmatizing attitudes of participants towards psychiatry and people with mental disorders [27]. This scale consists of 16 items, which are rated on 6 points from strongly agree to strongly disagree. The higher score suggests stronger stigma and more negative attitudes. We also used the Chinese version of MICA scale introduced by Li and tested with good validity and reliability (Cronbach's Alpha = 0.72) [28].

In order to further assess attitudes, participants were also asked to give their opinions towards two additional items: “People with severe mental disorders, such as schizophrenia, should stay a long time in hospital” and “It would be disgraceful for me if someone in my family had a serious mental disorder”. Items were rated on the 5-point Likert scale ranging from 1 = totally agree to 5 = totally disagree.

### Statistical analysis

Statistical analyses were conducted using IBM SPSS Statistics (Version 23.0; IBM Corporation, USA).First, descriptive statistics, including means and standard deviations (SD) for normally distributed variables and frequency and proportion for categorical variable, were calculated to describe differences on the demographic characteristics of CAWs. Second, analysis of variance (ANOVA) was used to compare differences of total scores of PDD, MICA and MAKS by different ages, care groups, and care willingness. Furthermore, Bonferroni corrections were conducted to analyze differences between any two groups if statistically significant differences were found by ANOVA. Last, multilinear regression models were used to calculate associations between MICA and age, care willingness, additional items and PDD total score among participants. To be specific, adjusted regression coefficients (*Ab*) of factors associated with MICA responses and corresponding 95% confidence intervals (*CI*) were calculated, while statistically significant demographics (age, educational level and types of CAWs) were included in the models. Significance was set at *P* < 0.05.

## Results

### Baseline characteristics

In total 384 CAWs were invited to participate in this study. As shown in Table 1, 381 care assistant workers completed our assessment. The response rate was 99.2% (381 of 384). Most CAWs were community cadres (52.2%), the rest of whom were community health workers (18.1%), community policemen (16.5%), volunteers (2.1%) and others (11.1%) who included patients’ relatives, friends and neighbors. It was found that 69.8% of CAWs were willing to deliver services towards patients with SMD, while 24.1% were neutral, and 6% were unwilling. All of these CAWs were responsible for delivering various kinds of services to people with mental disorders, for example, schizophrenia, schizoaffective disorder, bipolar disorder, paranoid mental disorders, or mental disorders due to epilepsy.

**Table 1:** Socio-demographic characteristics (baseline)

Characteristics	Participants (n = 381)
Age, years: mean (SD)	39.04 (9.07)
Education, years: mode (%)	15 (81.9)
Race, n (%)	
Han	376 (98.7)
Others	5 (1.3)
Sex, n (%)	
Male	227 (59.6)
Female	154 (40.4)
Religious, n (%)	
Atheists	363 (95.3)
Others	18 (4.7)
Care assistant workers, n (%)	
Community health workers	69(18.1)
Community policemen	63 (16.5)
Community cadres	199 (52.2)
Volunteers	8 (2.1)
Others	42 (11.1)
Care willingness, n (%)	
Willingness	266(69.8)
Neutral	92(24.1)
Unwillingness	23(6.0)

Data were indicated by mean, standard deviation (SD), frequency and proportion

### PDD, MAKS and MICA total scores

The mean total score was 36.45 (SD = 6.54) for the PDD, 22.72 (SD = 2.56) for the MAKS and 51.67 (SD = 7.88) for the MICA. Higher score indicated higher level of knowledge (MAKS) regarding mental health or higher stigma (PDD and MICA) towards mental disorders.

### Responses frequencies for additional items in the MICA scale

There were 2 additional items added to the MICA for further assessing participants' attitudes. One is "People with severe mental disorders, such as schizophrenia, should stay a long time in hospital". The other is "It would be disgraceful for me if someone in my family had a serious mental disorder". Attitudes composed of three dimensions, agree (strongly agree and partly disagree), neutral and disagree (strongly disagree and partly disagree). Table 2 shows the results. In our results, 52.8% of CAWs agreed with item 1, while 66.9% of CAWs disagreed with item 2.

**Table 2:** Attitudes towards 2 additional items of MICA (n, %)

	Attitude	n = 381	%
Item-1			
People with severe mental disorders, such as schizophrenia, should stay a long time in hospital	Agree	201	52.8
	Neutral	98	25.7
	Disagree	82	21.5
Item-2			
It would be disgraceful for me if someone in my family had a serious mental disorder	Agree	40	10.5
	Neutral	86	22.6
	Disagree	255	66.9

### PDD, MAKS and MICA total scores and socio-demographic variables

1. Comparison by age
  As shown in Table 3, there was no difference among the four-age groups in the scale of PDD or MAKS. However, there was a significant difference in the MICA score (*P* < 0.001). It demonstrated that CAWs who were 40–49 years old (53.58 ± 8.06) or 50–60 years old (55.13 ± 7.30) had significant higher MICA scores compared with CAWs with 20–29 years old (48.82 ± 7.01) and 30–39 years old (49.71 ± 7.33). It suggested that the older groups showed more stigma.
2. Comparison by types of CAWs
  As shown in Table 4, differences of the PDD, MAKS and MICA total scores on the four groups were all significant (*P* < 0.05). Community policemen had the highest score for PDD (38.33 ± 6.36) and MICA (55.63 ± 7.49), and community mental health staff had the lowest score in the MICA (48.16 ± 7.76). Community mental health staff and community policemen had higher MAKS scores (23.37 ± 2.65 for community policemen and 23.59 ± 2.69 for Community mental health staff) than those of community cadres (22.41 ± 2.63) and others (21.96 ± 2.24).
3. Comparison by willingness to provide care
  As shown in Table 5, there were significant differences in PDD (*P* < 0.05) and MICA scores among CAWs with difference care willingness (*P* < 0.001).CAWs with those who demonstrated attitudes of unwillingness (55.26 ± 9.69) or neutral (54.47 ± 6.85) had significant higher MICA scores than those who were willing to deliver care (50.39 ± 7.72).

**Table 3:** Differences of PDD, MAKS and MICA total scores among different age groups (Mean ± SD)

Measurements	Age groups				F	P
	20–29	30–39	40–49	50–60		
PDD	35.86 ± 5.68	36.16 ± 6.37	36.98 ± 7.24	36.60 ± 6.17	0.58	*0.63*
MAKS	22.54 ± 2.41	22.69 ± 2.66	22.91 ± 2.46	22.56 ± 3.39	1.87	0.60
MICA	48.82 ± 7.01	49.71 ± 7.33	53.58 ± 8.06*	55.13 ± 7.30*	*12.70*	* < 0.001*

^*^Compare with 20–29 age group and 30–39 age group, *P* < 0.05

**Table 4:** Differences of PDD, MAKS and MICA total scores among care assistant workers (Mean ± SD)

Measurements	Community mental health staff	Community policemen	Community cadres	Others	F	P
PDD	36.77 ± 7.76	38.33 ± 6.36	*35.51 ± 5.89*^*#*^	37.42 ± 6.88	3.61	0.013
MAKS	23.59 ± 2.69	23.37 ± 2.65	22.41 ± 2.63*	21.96 ± 2.24*^#^	*6.28*	* < 0.001*
MICA	48.16 ± 7.76	55.63 ± 7.49*	51.13 ± 7.49*^#^	53.66 ± 7.57*	*12.26*	*< 0.001*

^*^Compare with community mental health staff, *P* < 0.05; ^#^ Compare with community policemen,* P* < 0.05

**Table 5:** Differences of PDD, MAKS and MICA total scores among care willingness (Mean ± SD)

Measurements	Willingness	Neutral	Unwillingness	F	P
PDD	35.84 ± 6.57	37.67 ± 6.02	38.65 ± 7.34	*4.13*	*0.017*
MAKS	22.80 ± 2.64	22.48 ± 2.69	22.83 ± 2.69	0.51	0.60
MICA	50.39 ± 7.72	54.47 ± 6.85*	55.26 ± 9.69*	*12.41*	*< 0.001*

^*^Compare with willingness, *P* < 0.05

### PDD, MAKS and MICA total scores and additional items

As shown in Table 6, PDD total scores were significantly different for additional item 2 (affiliate stigma) (*P* < 0.001) rather than item 1 (views towards in patients) (*P* > 0.05). Scores of CAWs with attitudes of agreeing (40.13 ± 6.51) or being neutral (37.98 ± 5.50) towards additional item 2 were higher than that of who disagreed (35.36 ± 6.58). But MAKS total scores were significantly different for additional item 1 (*P* < 0.05), not for item 2 (*P* > 0.05). Scores of CAWs with attitudes which were neutral (22.42 ± 2.60) towards additional item 1 were lower than of those who disagreed (23.41 ± 2.56). However, MICA total scores were significantly different for both item 1 (*P* < 0.001) and item 2 (*P* < 0.001). For additional item 1, scores of CAWs with attitudes of agreement (53.40 ± 7.43) and neutral (51.46 ± 7.15) were higher than that of those who disagreed (47.66 ± 8.38). For additional item 2, scores of CAWs with attitudes of agreement (59.78 ± 4.62) or being neutral (55.06 ± 6.09) towards additional item 2 were higher than those who disagreed (49.25 ± 7.56).

**Table 6:** Differences of PDD, MAKS and MICA total scores on additional items (Mean ± SD)

Measurements	Agree	Neutral	Disagree	F	*P*
Item 1: People with severe mental disorders, such as schizophrenia, should long stay in hospital					
PDD	36.76 ± 6.67	36.17 ± 6.41	36.04 ± 6.42	0.48	0.621
MAKS	22.59 ± 2.68	22.42 ± 2.60*	23.41 ± 2.56	*3.75*	*0.024*
MICA	53.40 ± 7.43*	51.46 ± 7.15*	47.66 ± 8.38	*16.81*	*< 0.001*
Item 2: It would be disgraceful for me if someone in my family had a serious mental disorder					
PDD	40.13 ± 6.51*	37.98 ± 5.50*	35.36 ± 6.58	*12.93*	*< 0.001*
MAKS	22.78 ± 2.90	22.43 ± 2.50	22.81 ± 2.67	0.67	0.511
MICA	59.78 ± 4.62*	55.06 ± 6.09*	49.25 ± 7.56	*52.20*	*< 0.001*

^*^Compare with disagree, *P* < 0.05

### Association between MICA and other measurements among participants

As shown in Table 7, after controlling for sex, education level and CAWs, CAWs’ age (b_*ad*_ = 0.095, *P* = 0.012), care willingness (b_*ad*_ = 1.784, *P* < 0.001), and PDD total score (b_*ad*_ = 0.279, *P* < 0.001) were significantly positively associated with the MICA total score, while attitudes on additional item 1 (b_*ad*_ = − 0.8, *P* = 0.005) and attitudes on additional item 2 (b_*ad*_ = − 2.493, *P* < 0.001) were significantly negatively associated with the MICA total score. The R^2^ value of the multiple linear regression model was 0.454.

**Table 7:** Multivariate regression coefficients and 95% confidence intervals for the association between MICA and other measurements among participants

Characteristics	b (95% CI)	P	b_*ad*_ (95% CI)^a^	P
Age	0.240 (0.155 to 0.324)	< 0.001	*0.095 (0.021 to 0.169)*	0.012
Care willingness	2.609 (1.751 to 3.466)	< 0.001	1.784 (1.076 to 2.491)	< 0.001
Additional item 1	− 2.089 (− 2.720 to − 1.459)	< 0.001	− 0.8 (− 1.353 to − 0.247)	*0.005*
Additional item 2	− 3.514 (− 4.113 to − 2.916)	< 0.001	− 2.493 (− 3.061 to − 1.926)	< 0.001
PDD total score	0.451 (0.338 to 0.564)	< 0.001	0.279 (0.183 to 0.376)	< 0.001
MAKS total score	− 0.340 (− 0.638 to − 0.042)	*0.025*	–	0.101

b_*ad*_: Partial regression coefficient after controlling for confounding variables; CI: Confidence interval

^a^Adjusted for the participants’ sex, education level and CAWs

## Discussion

In recent years, the provision of care for people with mental disorders in China has developed from experience-based policies to evidence-based practices. The balanced care model increases service accessibility and task shifting building the human resource shortage in LMICs [13, 16, 29]. CAWs arise from special context of socio-political culture in China, mainly including community cadres, community mental health staff, community policemen. They are essential to bridge the human resources gap and delivering better mental health care in China. However, the quality of care delivered by CAWs could be influenced by their knowledge and attitudes related to mental health. As far as we know, this is the first study to investigate the mental health related knowledge and attitude of CAWs in the Chinese community, especially community policemen whose attitudes were previously unknown.

Policies on mental disorders in China have developed based on different characteristics of periods in history, from the “public prevention and public treatment” in the twentieth century to a social governance model—collaboration, participation and common interests in twenty first century. The human rights of people with SMD have been recognised and improved, which is consistent with the goals of the Comprehensive Mental Health Action Plan 2013–2020 [30]. CAWs play important roles in delivering better mental health services to people with SMD in the community, which could also be regarded as a manifestation of respect of human rights.

It is worth noting that CAWs in our study mainly provide mental health care to people with SMD, such as schizophrenia, schizoaffective disorder, paranoid mental disorders, bipolar disorder, epilepsy or mental retardation, which is consistent with the “686 Program” [19]. In this study, we found that negative attitudes towards patients with SMD were common among older age groups of staff, especially for those between 40–49 and 50–60 years old. It is consistent with previous studies which have found that middle and high ages have been one of the factors influencing attitudes towards people with mental disorders in high-income countries [31–33] and in China [24]. It is arguable that the middle-aged and elderly have witnessed different policies in Chinese history and have stereotypes which may have been formed during the long history of the mode of prevention and treatment management dominated by the large, specialized psychiatric hospitals.

These results also indicate that community policemen have relatively high level of knowledge but stronger negative attitudes towards people with mental disorders. This is a complex phenomenon. On the one hand, it has shown that negative attitudes to people with mental disorders are associated with lack of knowledge [34], on the other hand, the most policemen would not want a supervisor or manager who had a mental disorder [35], and this may reflect a particularly feature of police culture. However, community cadres had less stigma and discrimination compared with community policemen. We think the reason is related to "Task shifting", with the development of the economy and of society, meaning that more and more patients with SMD will be treated, rehabilitated and managed within the community to form a balanced service model between hospital and community service in mental health care [9]. Second, it is now within the roles and responsibilities of community cadres to manage people with mental disorders in China. Third, previous studies have found that it is common to find stigma and discrimination among community policemen [35], which is consistent with our results. But for community policemen, task shifting means that the functions of the police have been expanded from solving problems of violence to relatively less upon violence, but their original attitudes towards patients have not changed accordingly. Commonly they meet people with mental disorders during the course of their police work [36–39]. In fact, community policemen are playing important roles in providing care to people with mental disorders, including for patients at the onset of illness assessing potential violence, and assisting with involuntary hospitalization for patients with chronic illness. Therefore, it is notable that community policemen had a relatively high level of knowledge and understanding regarding stigma and other psychosis but more negative attitudes due to their job activities and unchanged perspectives. But for community cadres, things are different. Community cadres participate more in the rehabilitation of patients living in the community and have a frequent contact and further understanding towards them. Previous studies also showed that people who were familiar with and in close contact with patients with mental disorders tended to have a more positive attitude toward them [40–42] supporting the evidence for social contact as an effective intervention to reduce stigma.

CAWs usually share a similar community culture with patients with SMD, so their positive attitudes could help patients increase their accessibility to mental health care. Therefore, it is significant and necessary to assess and improve CAWs’ knowledge and attitudes towards mental disorders. Considering the socio-political culture in China, specific strategies aimed at reducing negative attitudes and stigma and discrimination should be carried out towards particular staff groups such as CAWs.

### Strengths and limitations

There are several limitations in this study which should be considered. First, this study aims to report the baseline level of attitudes and knowledge among CAWs, so it is not possible to know the causal relationship between attitudes towards people with SMD and related factors. Second, connecting quantitative research with qualitative research, such as formative work which is absent in this study, will be a more effective approach to investigate factors related to stigma and discrimination.

## Conclusion

The findings in our study suggest that negative attitudes towards people mental disorders among CAWs are common, especially among older staff and community policemen. However, the inadequate knowledge and negative attitudes of CAWs could be improved by delivering better mental health services and by addressing the treatment gap. Therefore, investigating the baseline level of level of mental health related to knowledge and attitudes among CAWs is beneficial in order to carry out an anti–stigma campaign in Guangzhou, China to improve negative attitudes and reduce stigma and discrimination.

## Data Availability

The data supporting the conclusions of this study are not publicly available. If any researcher show interest in data or materials access, they should contact the corresponding author on the reasonable request.

## Abbreviations

CAWs: CAWs
LMICs: Low‑ and middle‑income countries
SMD: Severe mental disorders
PTSA: Policy, training, service, and assessment
PDD: Perceived devaluation and discrimination scale
MICA: Mental illness: clinicians’ attitudes
MAKS: Mental health knowledge schedule
CI: Confidence intervals
SD: Standard deviation
